# Supplementary material for: Automatic Network Fingerprinting through Single-Node Motifs
Source: PLoS One. 2011 Jan 31;6(1):e15765. doi: 10.1371/journal.pone.0015765 (PMC3031529; doi:10.1371/journal.pone.0015765)
Supplement: File S1 — Supplementary figures, notes on software implementation, notes on run-time complexity, and detailed discussion of the small-world network results. (PDF) [file pone.0015765.s001.pdf]

Supporting Information

C. Echtermeyer[1], L. da F. Costa[2], F. A. Rodrigues[3], M. Kaiser[*1,4,5]

November 25, 2010

[1]School of Computing Science, Claremont Tower, Newcastle University, Newcastle-upon-Tyne NE1 7RU, UK
[2]Instituto de Física de São Carlos, Universidade de São Paulo, São Carlos, PO Box 369, 13560-970 São Carlos, São Paulo, Brazil
[3]Departamento de Matemática Aplicada e Estatística, Instituto de Ciências Matemáticas e de Computação, Universidade de São Paulo, São Carlos, PO Box 668, 13560-970 São Carlos, São Paulo, Brazil
[4]Institute of Neuroscience, The Medical School, Framlington Place, Newcastle University, Newcastle-upon-Tyne NE2 4HH, UK
[5]Department of Brain and Cognitive Sciences, Seoul National University, Seoul 151-746, Korea
[*]Corresponding author. Electronic address: m.kaiser@newcastle.ac.uk

Contents

1 Supplementary Figures **2**

2 Notes on the Software-Implementation **6**
2.1 Kernel-Bandwidth 6
2.2 Number of Singular Nodes w 6
2.3 Number of Motif Groups k 6

3 Run-Time Complexity **6**

4 A Small-World Emerging - Detailed Result Discussion **7**

List of Figures

S1 Run-time complexity of local network measures 2
S2 Re-wiring process generating a small-world network 2
S3 Graphical user interface for the BtA-workflow 3
S4 Contour plots of PDFs estimated using two different Gaussian kernels 4
S5 Density functions of 2D-Gaussian with different correlation 5

1 Supplementary Figures

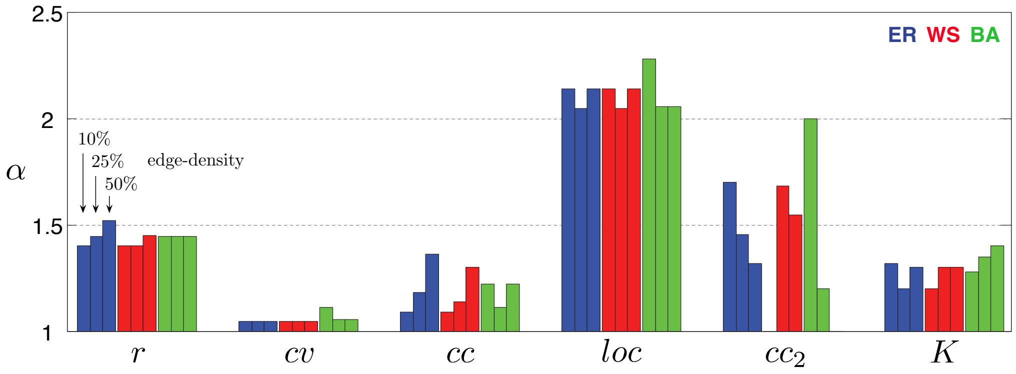

Figure S1: Run-time complexity $O(n^{\alpha})$ of the used local measures (r, cv, cc, loc, cc_2, K) increases polynomially in network size n (average value for 100 networks). Growth determining exponent α depends on edge-density (10%, 25%, 50%) and to lesser extent on network model (ER, WS, BA).

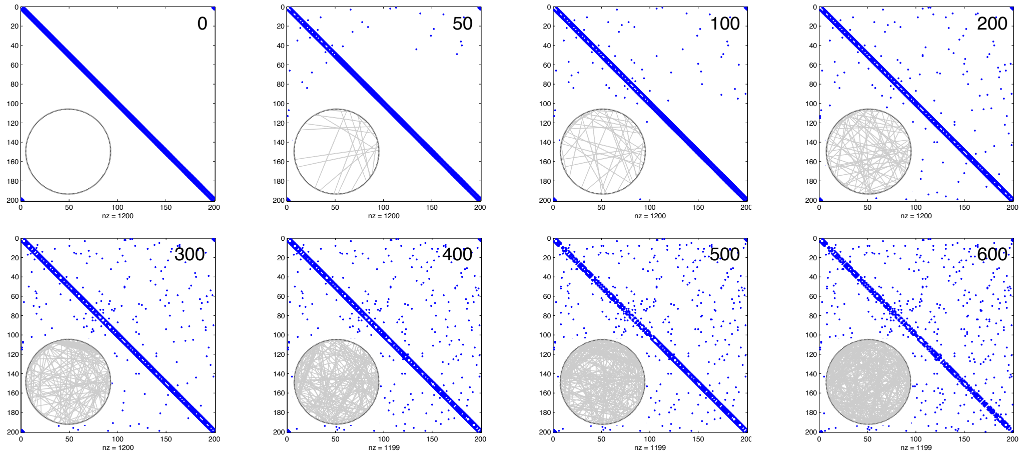

Figure S2: Adjacency matrix and belonging network during rewiring process as described by Watts and Strogatz [14] (number of steps in upper right corner; inset: network representation with nodes arranged on a circle). Beginning with a perfectly regular ring lattice (200 nodes) where each node is linked to its 6 closest neighbours (upper left), nodes are visited successively (one per step) and connections are randomly rewired with a probability of 40%. On the kth visit to a node, it is the link to the kth neighbour on the right, which is potentially rewired. After 600 steps (lower right) every node has been visited three times and on average 40% of all links have changed.

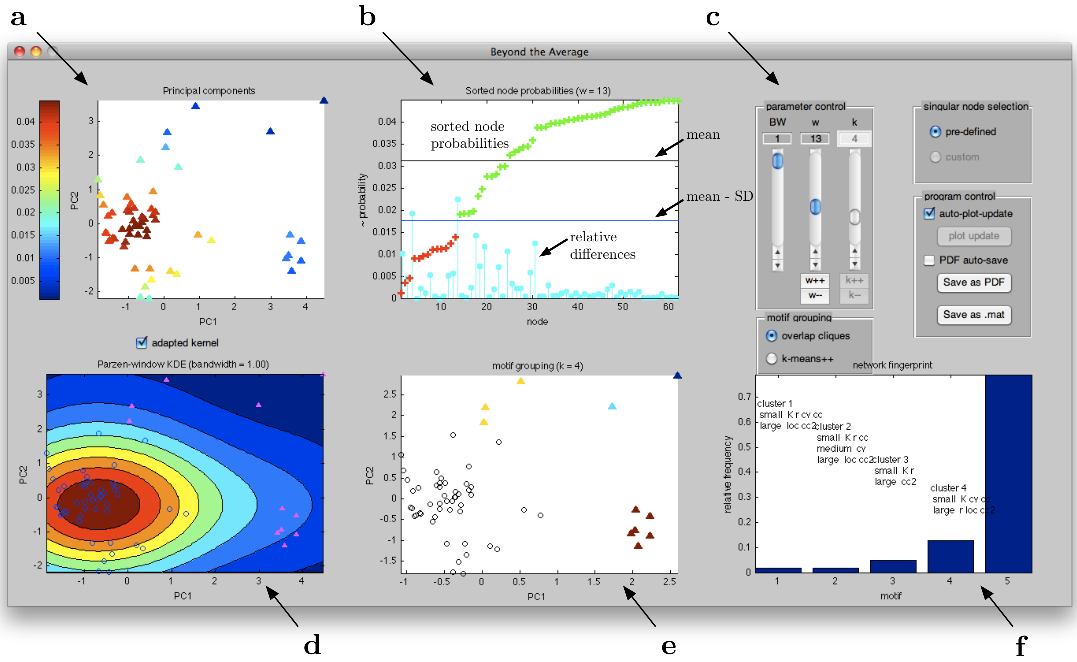

Figure S3: Graphical user interface for the BtA-workflow: **a** Nodes mapped to PCA-plane where their probability is coded by colour. **b** Sorted node probabilities and relative differences. Red and green colour indicates singular and regular nodes, respectively. Mean probability indicated by black line; blue line marks mean minus one standard deviation. Stems (cyan) indicate relative differences between their two adjacent probabilities. **c** Manual workflow-parameter control and options for result export. By default, changed settings show immediate effect in all plots (a,b,d–f). **d** Contour plot of PDF with reduced feature vectors superimposed, whose colour indicates whether they are classified regular or singular. **e** PCA-plane (rescaled by standard deviations) showing differently coloured motif groups. **f** Bar plot showing the relative frequency for each motif-region. A brief characterisation of each motif is given above its bar.

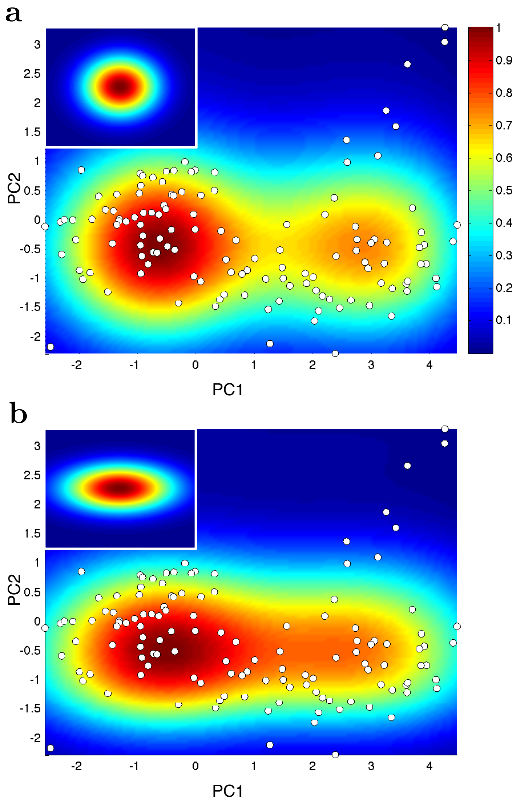

Figure S4: Contour plots of PDFs estimated using two different Gaussian kernels (upper left inlet). **a** Identical kernel bandwidth in both dimensions. With this symmetric kernel, the estimated PDF shows broad spreading of probability mass along vertical axis. **b** Kernel bandwidths scaled according to standard deviation along corresponding PC-axis. This adopted kernel results in a thinner and better matching PDF.

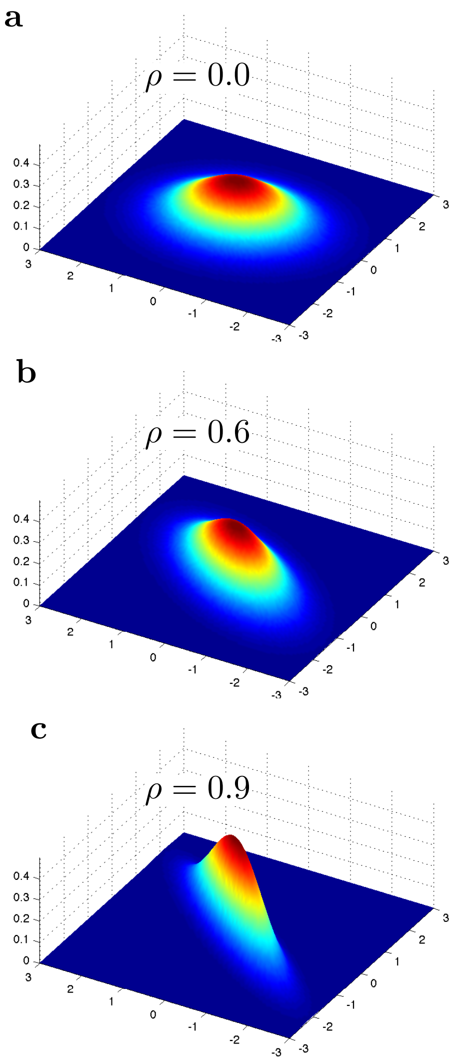

Figure S5: Density functions of 2D-Gaussian ($\sigma_x = \sigma_y = 1$) with different correlation ($\rho = 0.0, 0.6, 0.9$). **a** Uniformity of probability mass distribution around centre ($\mu_x = \mu_y = 0$) without correlation. **b** Gradually increasing correlation leads to tilting and **c** concentration of probability mass along the diagonal.

2 Notes on the Software-Implementation

Our implementation of the workflow including the automatic parameter determination is publicly available (`http://www.biological-networks.org/`). Below we briefly mention the workflow-alternatives of the software.

2.1 Kernel-Bandwidth

By default, the kernel-bandwidth is scaled according to the standard deviation along each principal component (PC) axis. Variability-based re-shaping of the kernel function improves the overall fit of the PDF to the points (Fig. S4). The kernel can also be made symmetric by deactivating the tick-box below the panel shown in Fig. S3a.

2.2 Number of Singular Nodes w

By default, our implementation of the workflow chooses the number of singular nodes w according to equation (1). This setting can be overwritten by the user, who is provided with a plot of all nodes' probabilities together with their relative differences (Fig. S3b). Manually chosen values for w can thereby be easily related to the default setting.

2.3 Number of Motif Groups k

Our implementation of the workflow provides 3 alternatives to determine k: By default motif-groups are determined deterministically through cliques of overlapping ellipses, as illustrated in Fig. 4. The user can also choose to determine the number of clusters using the ellipses, but perform clustering with k-means++. As the last alternative, k-means++ can be applied with a customised number of motif-groups.

3 Run-Time Complexity

The bulk of the runtime of the BtA-workflow is spent on step 1 where all selected local network measures are computed. Run-time complexity here depends on the measures that are chosen to characterise each node. We estimated how computational costs scale for six common local measures [4]. Like Costa et al. [5], we selected the normalised average degree r, the coefficient of variation of the degrees of the immediate neighbours of a node cv, the clustering coefficient cc [7, 14], the locality index loc, the hierarchical clustering coefficient of level two cc_2 [3], and the normalised node degree K. These measures have been applied to random networks, which have been generated according to the Erdős-Rényi (ER) [6], Watts-Strogatz (WS) [14], and Barabási and Albert (BA) [1] model. A polynomial function was fitted (root mean square error) to the average run-times to determine their dependence on network size. Additional to the size of the network, its edge density might also affect run-time, which is why we repeated the process while varying sparseness [1]. The results show relatively stable growth rates, irrespective of network model or connection density: Our naïve implementations of the six measures show run-time complexities ranging from linear to less than cubic (Fig. S1). Costs are thus comparatively cheap considering methods that identify specific connectivity patterns by counting occurrences of particular sub-graphs (e.g. [2, 8–11, 13]); such motif-counts also scale at least linearly in network size, but they show exponentially growing costs as the size of the motif-pattern increases [8]. In practice this often means that counts can not be determined for patterns involving 10 nodes or more [12], which renders some domains computationally intractable for this approach. In these cases the BtA-methodology might still be applicable: Local networks measures that only scale polynomially are comparatively fast to calculate and exceptional network characteristics can therefore even be identified in very large networks.

[1]For each random network-model (ER, BS, WS) any combination of network size ($n = 10, \ldots, 100$ nodes) and edge density (10%, 25%, and 50%) has been evaluated 100 times.

4 A Small-World Emerging - Detailed Result Discussion

In total we identified 5 singular node motifs, which differ in frequency and time of emergence (Fig. 3c): Motifs 2, 3 and 5 appear right from the beginning of the rewiring process; motifs 2 and 5 gradually become more common over time, whereas 3 levels out after a transient peak. The remaining motifs 1 and especially 4 only become apparent at later stages towards which both become more frequent. The motifs' temporally dependent expression levels can be understood by looking at their individual characteristics (Fig. 3d):

1. A node according to motif 1 has relatively few connections in contrast to its well connected neighbourhood. Other nodes that were initially linked to it have rewired themselves and because connections only change in 40% of the cases, motif 1 is rarely observed in early stages.

2. This contrasts the early appearance of motif 2 for which corresponding nodes are signified by many connections to a rather sparsely connected neighbourhood. From the starting point of a ring lattice such configuration occurs, as re-linking one of the initial regular connections destroys the local neighbourhood structure; if multiple nodes re-wire to the same target its degree grows, which makes the node a candidate for motif 2.

3. Motif 3-nodes have relatively few connections and nodes in their neighbourhood are similar in number of links and corresponding targets. This characterisation fits nodes linked to others that have been disconnected from the direct neighbours only. Such is likely to be observed during the first 200 steps of the rewiring process, where links to the closest neighbour are replaced, which is in agreement with motif 3's early peak in frequency. Later, when connections to further away neighbours are lost, the locality index decreases and fewer nodes fulfil the profile of motif 3.

4. The 4th motif mostly starts to appear when nodes are visited for the third time and some of the longest initial connections are replaced. At these late stages the ring lattice has undergone substantial perturbation, such that nodes differ widely in their degree and interconnectivity. Motif 4 describes rarely connected nodes whose neighbours have a diverse number of connections; but instead of being linked between each other, neighbours share other common targets.

5. The final motif 5 can be best characterised by its relation to the rest of the network, which shows a higher degree of connectivity than any node involved in the motif. Neighbours of the motif-node further vary in their number of connections and do not link to each other. This motif emerges early on, but its frequency rises more quickly during the last re-wiring-pass. During that time the last initial links are broken up and motif 5 emerges, as more parts of the network finally become sparse enough.

References

[1] Albert-László Barabási and Réka Albert. Emergence of Scaling in Random Networks. *Science*, 286(5439):509–12, 1999.

[2] Ilaria Bordino, Debora Donato, Aristides Gionis, and Stefano Leonardi. Mining Large Networks with Subgraph Counting. In *8th IEEE International Conference on Data Mining (ICDM)*. IEEE, 2008. ISBN 978-0-7695-3502-9.

[3] Luciano Da Fontoura Costa and Filipi Nascimento Silva. Hierarchical Characterization of Complex Networks. *Journal of Statistical Physics*, 125(4):841–76, 2006.

[4] Luciano Da Fontoura Costa, Francisco A. Rodrigues, G. Travieso, and P. R. Villas Boas. Characterization of complex networks: A survey of measurements. *Advances in Physics*, 56 (1):167–242, 2007.

[5] Luciano Da Fontoura Costa, Francisco A. Rodrigues, Claus C. Hilgetag, and Marcus Kaiser. Beyond the average: Detecting global singular nodes from local features in complex networks. *Europhysics Letters*, 87(July):18008, 2009.

[6] P. Erdös and A. Rényi. On Random Graphs I. *Publ. Math. (Debrecen)*, 6:290–7, 1959.

[7] Marcus Kaiser, Matthias Goerner, and Claus C. Hilgetag. Criticality of spreading dynamics in hierarchical cluster networks without inhibition. *New Journal of Physics*, 9:110, 2007.

[8] N Kashtan, S Itzkovitz, R Milo, and U. Alon. Efficient sampling algorithm for estimating subgraph concentrations and detecting network motifs. *Bioinformatics*, 20(11):1746–58, 2004.

[9] M Kuramochi and G Karypis. Frequent Subgraph Discovery. In *Proceedings of the 2001 IEEE International Conference on Data Mining*, pages 313–20. IEEE Computer Society, 2001. ISBN 0-7695-1119-8.

[10] Manuel Middendorf, Etay Ziv, and Chris H. Wiggins. Inferring network mechanisms: the Drosophila melanogaster protein interaction network. *Proceedings of the National Academy of Sciences of the United States of America*, 102:3192–7, 2005.

[11] R Milo, S. Shen-Orr, S Itzkovitz, N Kashtan, D Chklovskii, and U. Alon. Network motifs: simple building blocks of complex networks. *Science*, 298(5594):824–7, 2002.

[12] Pedro Ribeiro, Fernando Silva, and Marcus Kaiser. Strategies for Network Motifs Discovery. In *5th IEEE International Conference on e-Science*, pages 80–7. IEEE, 2009. ISBN 978-0-7695-3877-8.

[13] S. Wasserman and K. Faust. *Social Network Analysis: Methods and Applications (Structural Analysis in the Social Sciences)*. Cambridge University Press, 1994. ISBN 0521387078.

[14] Duncan J. Watts and Steven H. Strogatz. Collective dynamics of 'small-world' networks. *Nature*, 393:440–2, 1998.
